# Intracellular Delivery of Doxorubicin by Iron Oxide-Based Nano-Constructs Increases Clonogenic Inactivation of Ionizing Radiation in HeLa Cells

**DOI:** 10.3390/ijms22136778

**Published:** 2021-06-24

**Authors:** Roxana Cristina Popescu, Diana Iulia Savu, Miriam Bierbaum, Adriana Grbenicek, Frank Schneider, Hiltraud Hosser, Bogdan Ștefan Vasile, Ecaterina Andronescu, Frederik Wenz, Frank A. Giordano, Carsten Herskind, Marlon R. Veldwijk

**Affiliations:** 1 Department of Radiation Oncology, Universitätsmedizin Mannheim, Medical Faculty Mannheim, Heidelberg University, 68167 Mannheim, Germany; roxana.popescu@nipne.ro (R.C.P.); Miriam.Bierbaum@medma.uni-heidelberg.de (M.B.); Adriana.Grbenicek@medma.uni-heidelberg.de (A.G.); Frank.Schneider@umm.de (F.S.); Frank.Giordano@umm.de (F.A.G.); Carsten.Herskind@medma.uni-heidelberg.de (C.H.); 2 Department of Life and Environmental Physics, “Horia Hulubei” National Institute for Physics and Nuclear Engineering, 077125 Magurele, Romania; 3 Department of Science and Engineering of Oxide Materials and Nanomaterials, Politehnica University of Bucharest, 011061 Bucharest, Romania; Bogdan.vasile@upb.ro (B.Ș.V.); ecaterina.andronescu@upb.ro (E.A.); 4 Department of Anatomy and Developmental Biology, Center for Biomedicine and Medical Technology, Universitätsmedizin Mannheim, Medical Faculty Mannheim, Heidelberg University, 68167 Mannheim, Germany; Hiltraud.Hosser@medma.uni-heidelberg.de; 5 CEO, University Medical Center Freiburg, 79106 Freiburg, Germany; Frederik.wenz@uniklinik-freiburg.de

**Keywords:** radiosensitization, nanoparticles, iron oxide, polyethylene glycol, doxorubicin, drug delivery

## Abstract

In this study, we determined the potential of polyethylene glycol-encapsulated iron oxide nanoparticles (IONP_CO_) for the intracellular delivery of the chemotherapeutic doxorubicin (IONP_DOX_) to enhance the cytotoxic effects of ionizing radiation. The biological effects of IONP and X-ray irradiation (50 kV and 6 MV) were determined in HeLa cells using the colony formation assay (CFA) and detection of γH2AX foci. Data are presented as mean ± SEM. IONP were efficiently internalized by HeLa cells. IONP_CO_ radiomodulating effect was dependent on nanoparticle concentration and photon energy. IONP_CO_ did not radiosensitize HeLa cells with 6 MV X-rays, yet moderately enhanced cellular radiosensitivity to 50 kV X-rays (DMF_SF0.1_ = 1.13 ± 0.05 (*p* = 0.01)). IONP_DOX_ did enhance the cytotoxicity of 6 MV X-rays (DMF_SF0.1_ = 1.3 ± 0.1; *p* = 0.0005). IONP treatment significantly increased γH2AX foci induction without irradiation. Treatment of HeLa cells with IONP_CO_ resulted in a radiosensitizing effect for low-energy X-rays, while exposure to IONP_DOX_ induced radiosensitization compared to IONP_CO_ in cells irradiated with 6 MV X-rays. The effect did not correlate with the induction of γH2AX foci. Given these results, IONP are promising candidates for the controlled delivery of DOX to enhance the cytotoxic effects of ionizing radiation.

## 1. Introduction

The current standard multimodal treatment of most cancers consists in the surgical excision of the tumor and a combination of radiotherapy and chemotherapy [1]. In order to minimize systemic adverse effects, targeted therapies involving nanoparticle-based systems have been proposed [2]. In this context, the use of iron oxide nanoparticles (IONP) is a promising approach to improve the impact of conventional chemo- and radiotherapy in treating cancer. These nanoparticles have been clinically employed in imaging [3,4] and hyperthermia treatment [5,6] of different cancers.

Based on their size and magnetic properties, IONP have the ability to pass biological barriers and to be magnetically targeted towards the tumor site [7] where they can effectively deliver the active substance. In combination with radiotherapy, high-Z nanoparticles such as gold have been explored as radiosensitizers due to the release of high-intensity, low-energy electrons from their surfaces. IONP with intermediate Z can also potentially induce the production of secondary reactive species [8] which can lead to the alteration of metabolic function, DNA damage, protein expression, division or even to induction of cell death [9,10]. However, some nano-systems such as encapsulated IONP have the added advantage of being able to directly deliver cytotoxic or radiosensitizing compounds.

The sensitizing response is not only dependent on the physico-chemical characteristics of the used nanoparticles, such as composition [11,12,13], dimension [14,15], surface area [16,17], coating [17,18,19,20], but also influenced by their targeting ability and intracellular localization [14,17,21,22,23,24], as well as by radiation type and properties, such as energy, dose, and flow [17,25].

The aim of this study was to use core–shell polyethylene glycol-encapsulated IONP (IONP_CO_) to load the chemotherapeutic substance doxorubicin (DOX) (IONP_DOX_), in order to obtain radiosensitizing effects in human cervical adenocarcinoma cells (HeLa) at different doses and energies of the ionizing radiation. IONP are able to generate highly genotoxic reactive oxygen species due to the Fenton reaction and Haber–Weiss cycle [26]. Additionally, the combined effect of DOX with radiation has been previously employed clinically [27,28,29,30,31], its controlled delivery using nanoparticles promising to reduce the reported systemic toxic effects [32,33,34,35,36,37].

To the best of our knowledge, a drug delivery system based on iron oxide nanoparticles encapsulated in a polyethylene glycol shell has never been used before for radiosensitization purposes. In addition, drug-free polyethylene glycol-encapsulated iron oxide nanoparticles proved to have radiomodulatory effects in HeLa cells exposed to low-energy X-ray radiation treatment, which is reported here for the first time.

## 2. Results

**Figure 1:**
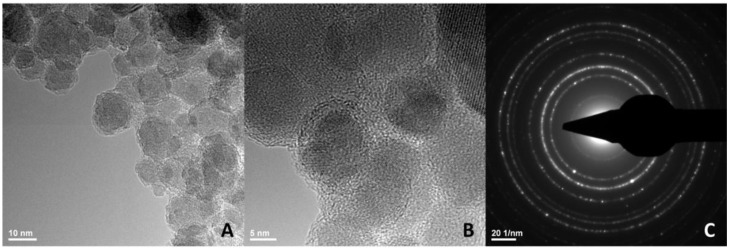
(**A**,**B**) High-resolution transmission electron microscopy (HR-TEM) at different magnifications and (**C**) selected-area electron diffraction (SAED) of IONP.

Polyethylene glycol-encapsulated iron oxide nanoparticles (IONP_CO_) were synthesized to encapsulate the anthracycline doxorubicin (IONP_DOX_). High-resolution transmission electron microscopy (HR-TEM) demonstrated that round crystalline nanoparticles with core–shell morphology were produced (Figure 1A,B). The crystallinity was confirmed by selected-area electron diffraction (SAED), which displayed diffraction rings characteristic of magnetite crystalline planes (Figure 1C): (220), (222), (400), (422), (333), (440). 

**Figure 2:**
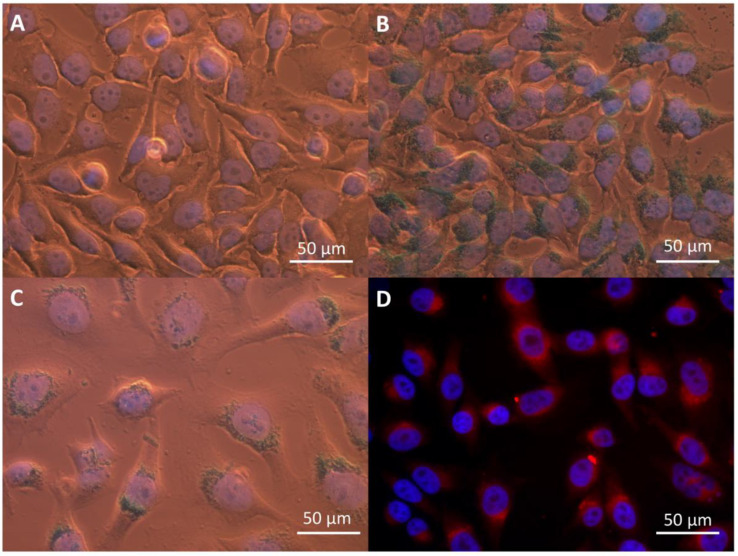
Internalization of IONP in HeLa cells: (**A**) control, (**B**) 100 µg/mL IONP_CO_, and (**C**,**D**) 100 µg/mL IONP_DOX_; (**A**–**C**) optical microscopy images, Prussian blue counterstaining of Fe and DAPI staining of nuclei; (**D**) fluorescence microscopy images, DAPI staining of nuclei (blue) and DOX auto-fluorescence (red).

**Figure 3:**
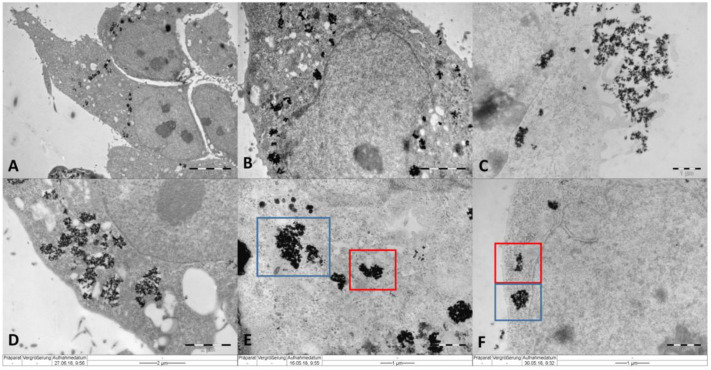
Internalization of (**A**) IONP_CO_, (**B**) IONP_DOX_ in HeLa cells in a peri-nuclear pattern; (**C**) macropinocytosis of IONP; (**D**) localization of IONP_CO_ in vesicle structures; (**E**,**F**) localization of IONP_DOX_ in the cytoplasm (blue square) and their exit from a vesicle structure (red square). The cells were incubated for 16 h with 100 μg/mL IONP.

The biological effects of IONP were determined for human cervical adenocarcinoma cells (HeLa). Optical, fluorescence, and electron microscopy confirmed the successful and efficient internalization of the IONP in HeLa tumor cells after 16 h of incubation, as well as their localization in the peri-nuclear area (Figure 2 and Figure 3), as described previously [38]. 

**Figure 4:**
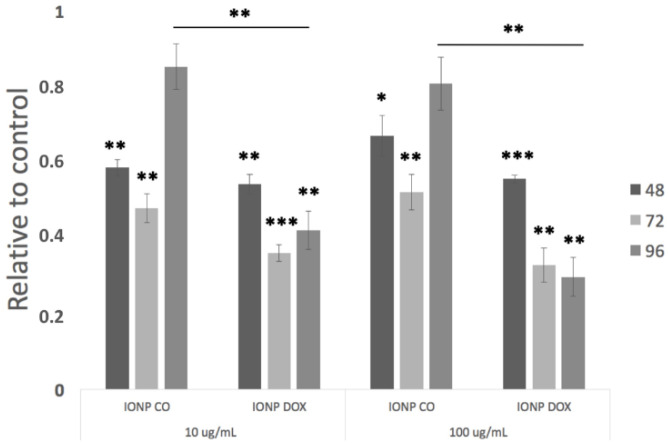
Proliferation of HeLa cells after incubation with different concentrations (10, 100 µg/mL) of IONP up to 96 h. Data are presented relative to untreated control cells and as mean ± SEM (n = 3); * *p* < 0.05, ** *p* ≤ 0.01 and *** *p* ≤ 0.001.

Analysis of proliferation (MTT assay; Figure 4) showed an initial (48/72 h) decrease in proliferation for all groups compared to untreated controls, irrespective of IONP concentration. While for IONP_CO_-treated cells this recovered to control levels 96 h post treatment, for IONP_DOX_-treated cells, this difference remained significant (*p* ≤ 0.01), irrespective of IONP concentration. 

**Figure 5:**
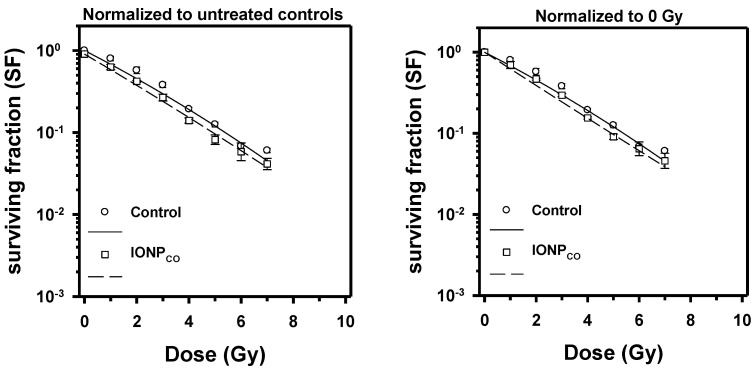
Clonogenic survival of HeLa cells after exposure to 100 μg/mL IONPs for 16 h, followed by 50 kV X-ray treatment. Data are presented as mean ± SEM (n = 3).

**Figure 6:**
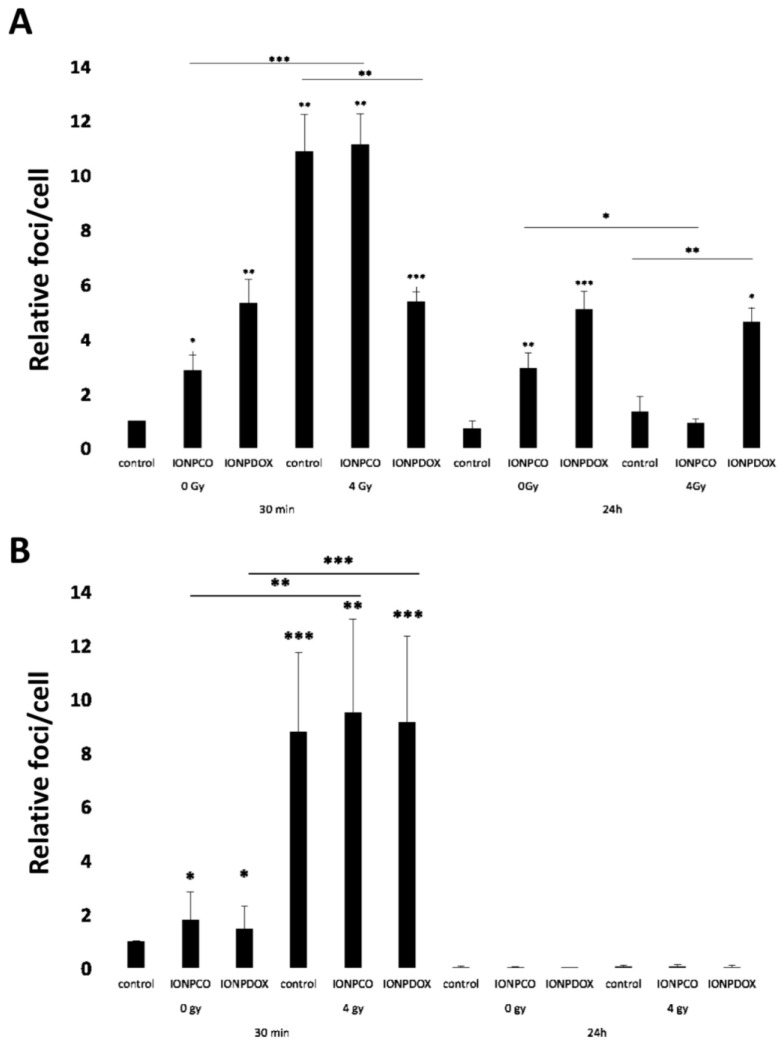
Double-strand DNA damage measured by γH2AX foci detection after 100 μg/mL IONP treatment for 16 h and/or 4 Gy and 50 kV (**A**) 6 MV (**B**) X-ray irradiation; the analysis was performed 30 min and 24 h after irradiation. Data are presented as relative foci per cell (versus the control group) and mean ± SEM (n = 3); * *p* < 0.05, ** *p* ≤ 0.01 and *** *p* ≤ 0.001.

The radiomodulating effect of IONP was investigated for low- (50 kV) and high- (6 MV) energy X-ray irradiation. We found that 50 kV X-rays with a mean photon energy in the range just above the K- absorption edge of iron (7.1 keV) induced a small but significant radiosensitizing effect of 100 µg/mL IONP_CO_ in HeLa cells (Figure 5). The dose-modifying factor calculated for a survival fraction of 0.1 was DMF_SF 0.1_ = 1.13 ± 0.05 (*p* = 0.01, n = 3). γH2AX repair foci (a surrogate marker for DNA double-strand break induction and repair) were increased 8.78 ± 2.91-fold (*p* < 0.001; 4 Gy control vs. 0 Gy control) 30 min after irradiation with a single dose of 4 Gy, and some residual foci remained at 24 h indicating that DNA double-strand break repair was not complete (NS; Figure 6A). Similarly, γH2AX foci were induced 30 min after 4 Gy of 6 MV X-rays (Figure 6B), although the level seemed slightly lower than for 50 kV, but in this case, few residual foci remained 24 h after irradiation (Figure 6A).

**Figure 7:**
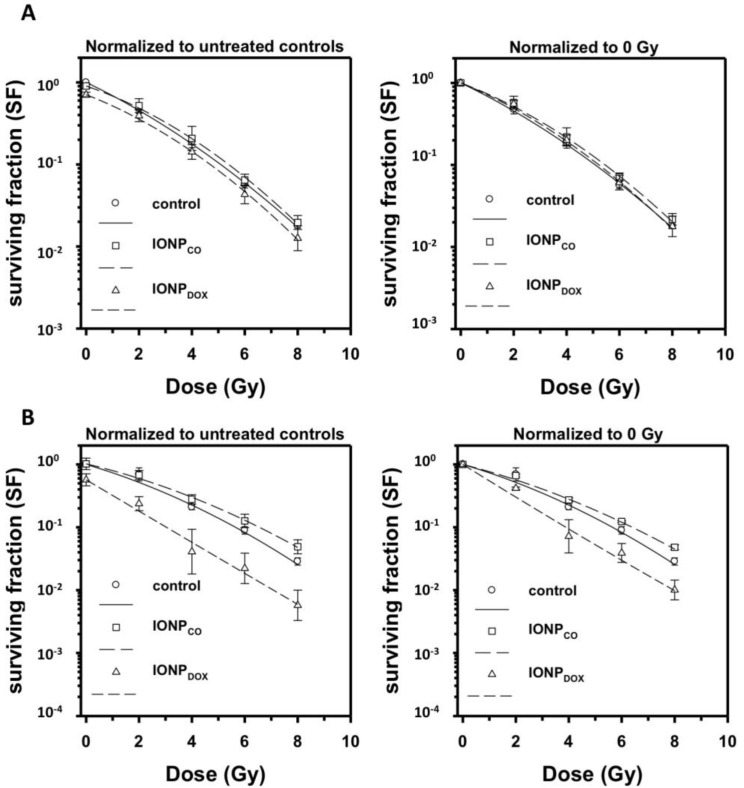
Clonogenic survival of HeLa cells after exposure to 10 µg/mL (**A**) and 100 μg/mL (**B**) IONPs for 16 h, followed by 6 MV X-ray treatment. Data are presented as mean ± SEM (n = 3).

IONP_CO_ treatment at 10 or 100 µg/mL followed by 6 MV X-rays yielded less steep survival curves characterized by a stronger downward curvature than for 50 kV X-rays. However, IONP_CO_ did not induce significant radiosensitization (Figure 7). 

Incubation with DOX-loaded IONP (IONP_DOX_) prior to radiation treatment induced a radiomodulatory effect dependent on the nanoparticle concentration (Figure 7). Treatment with 10 µg/mL IONP_DOX_ prior to irradiation with 6 MV irradiation did not result in a significant radiosensitization of the cells (Figure 7A). However, 100 µg/mL IONP_DOX_ caused a significant decrease in cell survival at 2 and 4 Gy (IONP_DOX_ vs. control: *p* = 0.009 (2 Gy) and *p* = 0.02 (4 Gy); Figure 7B). The dose-modifying factor of IONP_DOX_ previous exposure to radiation therapy was DMF_SF 0.1_ = 1.30 ± 0.10 (*p* < 0.001). 

IONP_DOX_ alone resulted in a significant induction of γH2AX foci, compared to control cells after 16 h of incubation (Figure 6). However, irradiation with either 50 kV (Figure 6A) or 6 MV (Figure 6B) X-rays negated this effect (30 min after irradiation). Residual foci were noticed in the case of cells treated with IONP_DOX_ and 50 kV; however, the effect was induced rather by nanoparticles than by radiation (IONP_DOX_ 0 Gy vs. IONP_DOX_ 4 Gy: NS, Figure 6B). This suggests that the reduced surviving fraction of HeLa cells treated with IONP_DOX_ might be due to an additive cytotoxicity of DOX when using 6 MV X-rays (IONP_DOX_ vs. IONP_CO_: *p* < 0.05 at all doses; Figure 7).

## 3. Discussion

In this study, we evaluated the radio-modulating effects of core–shell IONP_CO_ and IONP_DOX_ on human cervical adenocarcinoma (HeLa) cells after exposure to different doses and energies of ionizing radiation. In addition to the clinical relevance of cervical adenocarcinoma, as this type of cancer has a high frequency in the female population of developing countries [39], the HeLa cell line has been extensively used in studies involving novel nanomaterials for anti-cancer therapeutic purposes [15,40,41,42].

The proliferation results showed that DOX-free PEG-encapsulated iron oxide nanoparticles (IONP_CO_) were biocompatible for human cervical adenocarcinoma cells, with a maximum reduction of cell viability after 96 h of 19.19 ± 6.94% for 100 µg/mL (Figure 4). These observations were confirmed by determining the clonogenic survival of HeLa cells exposed for 16 h to different concentrations of IONP_CO_ (Figure 5 and Figure 7) and by literature data [43,44,45]. Such characteristic is mandatory and requested for radiomodulatory applications involving NPs [46]. 

Tumor cells were incubated for 16 h with IONP and then irradiated. This time of interaction between NPs and HeLa cells is shorter than one normal complete cell cycle and sufficient to prevent a possible dilution of internalized NPs due to cell division [47]. In addition, our previous results [38] showed high concentrations of nanoparticles internalized during this timeframe using concentrations of 100 μg IONP/mL: 31.66 ± 3.06 pg Fe_3_O_4_/cell in case of IONP_CO_ and115.2 ± 9.8 pg Fe_3_O_4_/cell for IONP_DOX_.

Both low- (50 kV) and high- (6 MV) energy X-ray sources were explored, as having different clinical relevance [48,49] and yielding a different amount of secondary electrons after their interaction with matter. It has been previously shown that the dose-modifying factor is higher at lower energies, and optimum radiosensitization occurs in the kV range [25,41,50,51,52]. Our results confirmed a moderate radiosensitizing effect of IONP_CO_ at 50 kV and no significant effect of 6 MV X-rays. These modest effects are consistent with Monte Carlo simulations [46] showing that iron oxide nanoparticles have a lower radiomodulating effect compared to other typically used high-Z nanoparticles, such as gold, gadolinium, or iodine. However, the dose-modifying efficiency increased with nanoparticles concentration and lowering of incident X-rays energies. Here, IONP_CO_ showed a radiosensitizing effect dependent on nanoparticle concentration and photon energy. Significant effects were noticed at the highest NP concentration (100 µg/mL) and lower energies (50 kV). To the best of our knowledge, there are no other studies involving polyethylene glycol-encapsulated iron oxide nanoparticles used for radiosensitization purposes involving conventional X-ray exposure.

Furthermore, the internalization and localization of the nanoparticles inside cells is a key factor in their radiomodulating efficiency. Considering that the path of secondary electrons is limited from tens of nanometers to few micrometers, depending on the nanoparticles physico-chemical properties and energy of radiation source [25], the radiosensitizing effect is highly dependent on nanoparticle localization inside a cell. Klein et al. [17] have shown that citrate- and malate-coated iron oxide nanoparticles not only have a better stability than bare NPs, but also have the ability to escape the vesicles within which they are internalized and to migrate through the cytoplasm into the endoplasmic reticulum, enhancing cells’ response to radiation treatment. Similarly, Hauser et al. [26] reported nanoparticles escaping lysosome membranes that directly influenced the normal functionality of the mitochondria by reactive oxygen production in the cytosol after irradiation. 

Incubation with 10 µg/mL IONP_DOX_ for 16 h prior to 6 MV X-ray exposure did not induce any significant alteration of the HeLa cells surviving fraction, but 100 µg/mL IONP_DOX_ followed by 6 MV X-ray irradiation resulted in a significantly increased clonogenic inactivation compared to both DOX-free IONP and control cells, with a DMF_SF 0.1_ = 1.30 ± 0.10 (excluding the intrinsic cytotoxicity of IONP_DOX_). These results suggest that the decrease in clonogenic survival measured after IONP_DOX_ treatment may be due to an interaction of the effects of irradiation with the intracellular released DOX, which is known to interact with and affect the genetic material of the cell and induce the DNA damage response. Our previous results [38] showed that most of the encapsulated DOX was delivered within 24 h, with a complete release at 70 h, independent of the medium pH. Using the same IONP concentration, larger amounts of IONP_DOX_ than IONPco were internalized by the cells. The effect correlated with the hydrodynamic diameter, which doubled after the loading of the drug. 

Other studies have also reported enhanced cytotoxic effects of ionizing radiation obtained using nanoparticle formula drug delivery systems [53,54,55,56,57]. Hamzian et al. [58] developed gemcitabine (GEM)-loaded poly (D, L-lactic-co-glycolic acid) (PLGA)-iron oxide nanoparticles for radiosensitization purposes using 0–7 Gy ^60^Co gamma irradiation. Their results showed that the drug delivery system and radiation treatment induced a significant decrease of tetrazolium salt metabolic ability in human breast cancer MCF-7 cells compared to only irradiated cells at 7 days after treatment. These results did indicate a reduced proliferation of the cells receiving both treatments, but no other investigation on the survival of these cells was done. No other reports on drug delivery systems based on polyethylene glycol-encapsulated iron oxide nanoparticles were previously published.

In the case of IONP_DOX_, the nanoparticles were internalized through macropinocytosis mechanisms, and a high amount appeared free in the cytoplasm, eventually located in the peri-nuclear area (Figure 3B,C,E,F). These NPs are organized in clusters of 160 nm composed of about 20 nm-PEG individually covered iron oxide cores (Figure 1). Similarly, other studies reported significant radiosensitization for NPs organized in clusters rather than single nanoparticles [26,40,58,59].

One of the direct effects of ionizing radiation consists in the alteration of DNA integrity, and γH2AX foci are an indirect marker of DNA double-strand breaks [60]. Our results showed that both IONP_CO_ and IONP_DOX_ increased the number of γH2AX foci in HeLa cells without X-ray exposure (Figure 6). As expected, ionizing radiation caused γH2AX induction, the previous treatment with both IONP_CO_ and IONP_DOX_ causing no significant effect compared to radiation alone 30 min after radiotherapy (Figure 6). It should be noted that some of the differences observed between the 50 kV and the 6 MV X-ray γH2AX data are most likely related to the difference in the seeding/irradiation procedure between the two groups (see materials and methods section). 

Similar observations were made by Stefancikova et al. [61] who used gadolinium-based nanoparticles for the radiosensitization of glioblastoma cells and showed that the effects correlated with lysosome disintegration caused nanoparticles liberation into the cytoplasm. Our observations on IONP_DOX_ behavior in HeLa cells, considering the internalization and localization of the NPs in the cytoplasm of the cells in the peri-nuclear area, the diminished clonogenic survival following additional radiation exposure, and the ability of the cells to repair the genome following both treatments, indicate that the cytotoxicity mechanisms of IONP_DOX_ and radiotherapy are beyond the induction of direct DNA damage.

## 4. Materials and Methods

### 4.1. Nanoparticle Synthesis and Characterization

Core–shell iron oxide nanoparticles encapsulated in polyethylene glycol (molecular weight 6 kDa) were generated using a two-step co-precipitation method as described previously [38]. Bare iron oxide nanoparticles were synthesized using a chemical co-precipitation method at room temperature. The resulting nanoparticles were encapsulated in polyethylene glycol 6000 Da using a 1:1 polymer/nanoparticles ratio, in an anhydrous medium [38]. 

The resulted nano-constructs (IONP_CO_) with a hydrodynamic diameter of 164.2 nm (polydispersity index (PDI) of 0.233 and zeta potential of 14.8 mV), were used to load the chemotherapeutic doxorubicin (DOX, IONP_DOX_), resulting in nanoparticles with a mean diameter of 369.1 nm (PDI 0.238 and zeta potential of −20.9 mV) [38]. The loading of the drug was carried out in an aqueous solution overnight, through ad/absorption [38]. The loaded quantity was 1.11 wt% DOX [38]. 

The nanoparticles were characterized using a Tecnai G2 F30 S-TWIN HR-TEM (Thermo Fisher Scientific, Hillsboro, OR, USA), equipped with a selected-area electron diffraction (SAED).

### 4.2. Cell Culture

The biological evaluation of the IONP was performed in human cervical adenocarcinoma cells HeLa (obtained from the Tumor Cell Bank of the German Cancer Research Center (DKFZ, Heidelberg, Germany)), which were cultured in Dulbecco’s modified Eagle’s medium (DMEM; Biochrom, Merck Millipore, Darmstadt, Germany) supplemented with 10% FBS (Biochrom, Merck Millipore, Darmstadt, Germany). Cell cultures were maintained at 37 °C in a humidified incubator (95% air, 5% CO_2_). The authenticity of the cell line was confirmed using STR profiling (100% identical to the ATCC STR database; Cell lines service GmbH, Eppelheim, Germany).

### 4.3. Treatment of Cells with IONP

HeLa cells at different concentrations were seeded and incubated for 4 h to allow their attachment. After this time, the culture medium was replaced with IONP-containing fresh medium (10, 100 µg/mL nanoparticles), and the cells were incubated for another 16 h.

### 4.4. Detection of IONP Internalization

2 × 10^5^ HeLa cells/well were seeded in 6-well plates and treated as described above. Following the incubation with IONP, the cells were washed several times with PBS, seeded on 10 mm glass slides, and allowed to adhere for another 24 h. After this period of time, the cells were fixed with 3.7% PFA. IONP were stained with Prussian blue (1:1 solution of 1 M HCl and 5% potassium ferrocyanide), at 37 °C for 15 min. The visualization was done using optical microscopy, while imaging was performed by fluorescence microscopy taking advantage of DOX native fluorescence. DAPI counterstaining of nuclei was applied. 

For the electron microscopy imaging, the cells were fixed with 3.7% PFA for 24 h immediately after IONP treatment. Then the cells were detached with a cell scraper and collected in PBS. Staining was performed using a 2% OsO_4_ solution (Plano GmbH, Wetzlar, Germany) for 1 h, and dehydration of the cells using ethanol and acetone (Carl Roth GmbH&Co. KG, Karlsruhe, Germany) solutions at increasing concentrations. Embedding of the samples was done in Epon (SERVA Electrophoresis GmbH, Heidelberg, Germany). 90 nm slices were placed on copper grids (Plano GmbH) and counterstained with uranyl acetate (SERVA Electrophoresis GmbH) followed by lead citrate (Merck KGaA, Darmstadt, Germany). Imaging of IONP-treated HeLa cells was performed using a Zeiss EM 10 transmission electron microscope (ZEISS, Oberkochen, Germany), equipped with an Olympus Megaview G2 camera (Olympus Europa SE & Co. KG, Hamburg, Germany).

### 4.5. Proliferation Assay

The proliferation of HeLa cells following IONP treatment was measured using the MTT tetrazolium-salt viability assay (Sigma–Aldrich Chemie GmbH, Darmstadt, Germany). Cell suspensions at different concentrations (3 × 10^3^, respectively 1.5 × 10^3^ cells/well) were put in 96-well plates avoiding confluency. The IONP treatment was applied as described above, and the cells were incubated during 48, 72, and 96 h. After each time point, the cells were incubated for another 2 h in presence of 10 µg/mL MTT (5 mg/mL in PBS), and the resulting formazan crystals were solubilized using DMSO. The absorbance of each corresponding sample was measured at 570 nm using a Tecan infinite M200 microplate reader (Tecan Group Ltd., Männedorf, Switzerland).

### 4.6. In Vitro Irradiation

50 kV X-rays were generated using a miniature x-ray source (PRS400 of the Intrabeam^®^ system; 50 kV/40 mA) equipped with a 4 cm spherical applicator for tumor-bed irradiation (Zeiss Surgical GmbH, Oberkochen, Germany). The dose rate distribution at the cell plane (0.49 Gy/min) in water-equivalent phantom [62] was determined by film dosimetry (GafChromic EBT, Wilmington, NJ, USA). 

6 MV X-rays were obtained from a clinical linear accelerator (Versa HD, Elekta Synergy, Stockholm, Sweden) at a dose rate of 6.67 Gy/min, using a 40 × 40 cm^2^ irradiation field. The samples were irradiated at a 100 cm source–surface distance with 15 mm water-equivalent material for dose buildup and 8 cm for backscatter. Dosimetry was performed by the staff medical physicists of the radiotherapy department as part of the daily quality checks.

### 4.7. Clonogenic Survival Assay

Samples for the colony formation assay (CFA) were prepared by seeding 10^5^ HeLa cells/well in 24-well plates. IONP treatment was done as described above. After the incubation time, the cells were carefully washed 3 times with PBS. Cells were detached, and different dilutions of each sample were made (200–5000 cells/T_25_ flask). Following irradiation, the cells were seeded at their respective densities using 5 mL of complete culture medium and incubated in standard conditions of temperature and humidity for 14 days. After this time, the samples were fixed using a methanol and acetic acid solution and stained using crystal violet, as reported [62]; counting and scoring was done as previously described [40]. The surviving fraction (SF) was fitted using a linear-quadratic model (ln(SF) = −(αD + βD^2^)), using the non-linear regression tool of SigmaPlot 11 (Systat Software GmbH, Erkrath, Germany) [40].

### 4.8. γH2AX Foci Detection

For the 6 MV radiation treatment, 2 × 10^5^ cells/well were seeded in 8-well chamber slides (Falcon, Corning Life Sciences, Amsterdam, The Netherlands) and treated with IONP as described above. The cells were then carefully washed 3 times with PBS and covered with 200 μL of nanoparticles-free culture medium. In the case of irradiation with 50 kV X-rays, samples were prepared as for the clonogenic survival assay and irradiated with 0 and 4 Gy. Cells exposed to 50 kV X-rays were attached to the slides using cytospin centrifugation (Thermo Shandon Cytospin 3, Walthman, MA, USA) immediately after irradiation. Fixing was done after 30 min, 24 h from irradiation using 3.7% PFA; non-specific binding was reduced by blocking with 1% BSA in PBST for 10 min; afterwards, the cells were incubated with anti-γH2AX antibodies (Abcam, Cambridge, UK) for 1 h, at room temperature; donkey anti-mouse FITC-labelled secondary antibodies (Abcam, Cambridge, UK) were used for visualizing γH2AX foci. Images were acquired using a Leica DMRE microscope equipped with a Leica DFC3000G camera, and counting was performed manually.

### 4.9. Statistical Analysis

All experiments were performed at least in triplicate, and data are presented as mean ± SEM, unless otherwise noted. Statistical analysis was done using a two-sided Student *t*-test, and one- and two-ways ANOVA (SigmaPlot 11, Systat Software GmbH, Erkrath, Germany).

## 5. Conclusions

This study evaluated the effect of both DOX-free and DOX-loaded polyethylene glycol-encapsulated iron oxide nanoparticles on the clonogenic survival of human cervical adenocarcinoma (HeLa) cells after exposure to low- (50 kV) and high- (6 MV) energy X-rays. IONP_CO_ proved a radiosensitizing effect dependent on NPs concentration and energy of the X-rays. IONP_CO_ showed a significant radiosensitizing effect with 50 kV X-rays. Incubation with 100 μg/mL IONP_DOX_ prior to 6 MV X-ray exposure resulted in a significantly increased clonogenic inactivation compared to both IONP_CO_ and control cells. The observed radiomodulatory effect of the IONP was not related to changes in DNA double-strand break induction. These results showcase the potential of IONP in bimodal chemo- and radiotherapy of tumor cells.
